# Placental lipase expression in pregnancies complicated by preeclampsia: a case–control study

**DOI:** 10.1186/s12958-015-0098-9

**Published:** 2015-09-04

**Authors:** Helen L. Barrett, Marta H. Kubala, Katherin Scholz Romero, Kerina J. Denny, Trent M. Woodruff, H. David McIntyre, Leonie K. Callaway, Marloes Dekker Nitert

**Affiliations:** UQ Centre for Clinical Research, The University of Queensland, Herston, QLD Australia; Obstetric Medicine, Royal Brisbane and Women’s Hospital, Herston, QLD Australia; School of Medicine, The University of Queensland, Herston, QLD Australia; Mater Research Institute, The University of Queensland Brisbane, Brisbane, QLD Australia; School of Biomedical Sciences, The University of Queensland, St Lucia, QLD Australia

**Keywords:** Preeclampsia, Intrauterine growth restriction, Lipase, Placenta, Pregnancy

## Abstract

**Background:**

Preeclampsia (PE) is associated with maternal and neonatal morbidity and mortality. In PE, the physiological hyperlipidaemia of pregnancy is exaggerated. The purpose of this study was to examine the expression of adipose triglyceride lipase (ATGL), hormone sensitive lipase (HSL), lipoprotein lipase (LPL) and endothelial lipase (EL) in pregnancies complicated by PE.

**Methods:**

Placentae were collected from 16 women with PE and 20 women with uncomplicated pregnancies matched for maternal prepregnancy BMI and gestational age of delivery. Gene and protein expression of the placental lipases were measured by Q-PCR and Western blot. DNA methylation of the promoter of LPL was assessed by bisulfite sequencing. Lipase localisation and activity were analysed.

**Results:**

Gene expression of all lipases was significantly reduced, as was HSL protein level in women with PE. All lipases were localised to trophoblasts and endothelial cells in PE and control placentae. There was no difference in methylation of the LPL promoter between PE and control placentae. Lipase activity was not altered in placentae from women with PE.

**Conclusion:**

These results suggest that the decreased placental lipase gene but not protein expression or lipase activity, which is associated with late-onset PE is not a major contributor to the abnormal lipids seen in PE.

## Background

Preeclampsia (PE) occurs in ~ 5 % of pregnancies in the developed world. During pregnancy, PE is associated with maternal multiorgan dysfunction, placental abruption and poor fetal growth. In the longer term, PE predicts maternal hypertension and carries an increased maternal cardiovascular and renal morbidity [1, 2]. One adverse infant outcome associated with PE is intrauterine growth restriction (IUGR), a failure of the infant to reach its full potential growth [3]. IUGR is associated with perinatal morbidity and mortality and also with hypertension and cardiovascular disease later in life for the infant [4, 5].

In PE, most studies report exaggerated and early maternal gestational hyperlipidaemia [6–8], with marked hypertriglyceridemia, higher very low density lipoprotein (VLDL) concentrations [9] and higher levels of small, dense low density lipoprotein (LDL) [10]. There is also a rise in maternal free fatty acid (FFA) concentrations above normal pregnancy levels [11]. This excessive increase in maternal lipids is thought to contribute to endothelial dysfunction, one of the hallmarks of PE [12, 13]. PE is also associated with abnormalities in lipid oxidation, which may be part of the underlying pathophysiology of the condition [12, 13]. Women with a history of PE demonstrate persistent abnormalities in lipids postpartum [14, 15].

The placenta supplies fatty acids (FFA) and cholesterol to the infant. While FFAs can diffuse across the placenta, most lipids require active placental transport through the activity of lipoprotein receptors, lipases and fatty acid binding proteins [16, 17]. Abnormal maternal lipids or altered placental lipid processing could contribute to the altered infant growth and change in cord lipoproteins seen in PE. The expression and activity of lipoprotein lipase (LPL) has been variably found to be higher, lower or unchanged in placentae from women with PE or IUGR compared to uncomplicated pregnancy [18–26]. Endothelial lipase (EL) gene expression has been reported to be decreased in IUGR [19] but has not been analysed in PE. The expression and localization of the intracellular lipases adipose triglyceride lipase (ATGL) and hormone sensitive lipase (HSL) have not previously been examined in placentae from women with pregnancies complicated by PE or IUGR.

The current study aims to examine placental lipase expression in late-onset PE. Placental gene, protein expression and localization of ATGL, HSL, EL and LPL was analysed in placentae from women with PE and IUGR and uncomplicated pregnancy. Furthermore, DNA methylation of the promoter of LPL was investigated and overall lipase activity was examined.

## Methods

### Subjects

Pregnant women in the third trimester were recruited from a tertiary general and obstetric hospital. All women gave written informed consent. Permission for the study was granted by the Human Research Ethics Committees of the Royal Brisbane and Women’s Hospital and The University of Queensland. Diagnosis of PE was defined by current Society of Obstetric Medicine, Australia New Zealand guidelines research definition [27]. Participants were matched for maternal BMI, which was calculated from a recorded early pregnancy weight in kg divided by the squared height in meters. The customized birth centile was calculated with the online calculator gestation.net (www.gestation.net). Small for gestational age (SGA) infants were defined as adjusted birth weight centile < 10^th^. Placental tissue was collected immediately post-delivery, sampled randomly (~ 1 cm^3^) but away from areas of infarction or calcification, snap-frozen in liquid nitrogen and kept at -80^0^ C until analysis. In addition, 1 cm^3^ samples of placenta for paraffin embedding were washed in PBS, placed into 4 % paraformaldehyde for 48 hours and kept in a saturated sucrose solution until embedding.

### Ethics approval

This study was approved by the Human Research Ethics Committees of the Royal Brisbane and Women’s Hospital (HREC/08/ARBW/16: 19/01/2009) and The University of Queensland (2009000115: 04/02/2009).

### RNA isolation and quantitative real-time PCR

Placental tissue was lyzed by violent shaking for 2 × 2 minutes at 30 Hz with a 5 mm stainless steel bead in a TissueLyser (Qiagen, Chadstone, VIC, Australia). mRNA was isolated from placenta with the Allprep RNA/DNA extraction mini kit (Qiagen). RNA was quantified by Nanodrop and all samples had 260/280 ratios > 1.8. 750 ng mRNA was reverse transcribed to cDNA with the QuantiTect reverse transcription kit (Qiagen) using an equal mixture of oligodT and random primers. Quantitative real-time PCR was performed on 18.75 ng of cDNA with 300 nM of primers and iTaq universal SYBR green mastermix (Bio-Rad, Gladesville, NSW, Australia) on an iQ5 PCR machine (BioRad). The PCR protocol consisted of 1 cycle at 95 °C for 10 min, 40 cycles of 95 °C for 15 sec and 59 °C for 1 min followed by dissociation curve analysis. Primers unique for the target gene and covering exon-exon junctions were designed with primerBLAST. The primer sequences are presented in Table 1. To adjust for potential differences in cellular composition of the placental samples, gene expression was normalized to the geometric mean of expression of the housekeeping gene TATA-box binding protein (*TBP*), cytokeratin 7 (*CK7*) as a marker for trophoblast cells, CD34 (*CD34*) for endothelial cells and desmin (*DES*) for smooth muscle cells. The analysis was also performed normalizing to the expression of *TBP* only yielding similar results.

**Table 1:** Primer sequences

Gene name	Forward primer	Reverse primer
*LPL*	5′-TGGATCGCTCCACTTTGACC	5′-GGGCTTCGGACTGGTAAACA
*LPIG*	5′-GTCCAGCCCCTGCTATCTCA	5′-CCTTTTCAAACTGACCCTTGCC
*LIPE*	5′-CACATTAGACCCAGAAGATGCC	5′-GGCAGCGAAACTTGACAGTG
*PNPLA2*	5′-TGCCCACTTTGTGTGTATGTG	5′-CCAGGAGTGCGACGCT
*CK7*	5′- CCGTGCGCTCTGCCTATGGGG	5′- GCTCCAGAAACCGCACCTTGTCGAT
*CD34*	5′- CCACAGGAGAAAGGCTGGGCGA	5′- AGCCCCTCGGTTCACACTGGC
*DES*	5′- TCCGAGAAACCAGCCCTGAGCAA	5′- GTGGCCTCACTGACGACCTCCC
*TBP*	5′-GGGCACCACTCCACTGTAC	5′-CTGTTCTTCACTCTTGGCTCCT

### Protein expression

Placenta were lysed with a RIPA buffer consisting of 50 mM Tris, 1 % Triton-X, 0.1 % SDS, 0.5 % DOC, 150 mM NaCl, and protease inhibitor cocktail (Roche, Applied Science, VIC, Australia). Tissue was disrupted by violent shaking for 2 × 2 minutes at 30 Hz with a 5 mm stainless steel bead in a TissueLyser (Qiagen). After lysis, the sample was centrifruged for 10 min at 4 °C and the protein content in the supernatant determined by bicinchoninic acid assay (Sigma-Aldrich, Castle Hill, NSW, Australia). 30 μg of protein was loaded onto a 4–12 % gradient NuPAGE® Bis-Tris gel (Life Technologies, Mulgrave, VIC, Australia), transferred onto a polyvinylidene difluoride (PVDF) membrane (Millipore, Kilsythe, VIC, Australia) and blocked for 1 hour with 5 % non-fat dry milk in PBS-Tween. Primary antibody for rabbit anti-LPL (1:300, sc-32885 Santa Cruz Biotech, Texas, USA), rabbit anti-HSL (1:150, sc-25843 Santa Cruz Biotech), rabbit anti-EL (1:150, 100030 Cayman chemical, Michigan, USA), or rabbit anti-ATGL (1:300, 2138 Cell Signalling Technology, Massachusetts, USA were co-incubated with mouse anti-β-Actin (1:20000, A5316, Sigma Aldrich) overnight at 4 °C with agitation. Secondary LI-COR antibodies, goat anti-rabbit 800CW (1:10000, 926–32211, LI-COR) and donkey anti-mouse 680LT (1:15000, 926–68022, LI-COR) were incubated for 1 hour at room temperature and protein was detected by the Odyssey Infrared Imaging System (LI-COR). Lipase protein expression was analyzed by densitometry correcting for differences in protein loading by using β-actin levels.

### Immunohistochemistry

Paraffin-embedded sections (5 μm) were baked, and rehydrated. Antigen retrieval was performed by heating to 125 degrees °C in 100 mM sodium citrate, 0.05 % Tween 20 at pH 6.0 for 30 minutes. Endogenous peroxidase activity was blocked with hydrogen peroxide 3 % for 10 mins followed by 15 mins with Biocare Background Sniper (MACH2, Biocare Medical, Concord CA). Immunolabelling was performed using polyclonal rabbit antibodies to LPL, anti-HSL, anti-EL antibodies (Biorbyt, Cambridge, UK: LPL (1:1000, orb13546), EL (1:500, orb100394), HSL (1:100, orb40070)) and ATGL antibody (1:100, Cell Signalling Technology). Confirmatory immunohistochemistry for LPL was performed with a second polyclonal rabbit antibody (1:1000, Santa Cruz Biotech, sc-32885). After washing, the slides were incubated with a biotinylated polyvalent goat secondary antibody followed by DAB incubation for 1 minute. Slides were counterstained with Harris’ Haematoxylin (HHS 16, Sigma Aldrich) and mounted with coverslips.

### Image analysis

HSL protein expression was analyzed with a quantitative immunohistochemistry method described by Helps et al. [28]. This method uses Ruifrok and Johnston's color deconvolution image processing method to digitally separate hematoxylin and DAB staining. The imaging processing and analysis was performed in NIH-ImageJ software using Landini’s ImageJ plugin then histogram analysis and a weighting calculation to estimate the amount of DAB staining. We took 10 randomly selected frames from each of 4 control and 4 PE placentae that were processed and stained concurrently. Within each frame, the placental villi were demarcated by hand on the NIH-ImageJ software.

### Lipase activity

Three milligrams of tissue was homogenized in 300 uL of ice cold assay buffer (150 mM NaCl, 10 mM Tris, 2 mM EDTA, pH 7.4) for 4 mins at 30Hz with a stainless steel bead using the Tissue lyser II (Qiagen). Homogenates were centrifuged for 10 mins at 10000 X g at 4 °C. Lipase enzyme activity was measured in supernatants using a commercial kit (Roar Biomedical, New York, USA) according to the manufacturer’s instructions. LPL activity in the supernatant was measured in duplicate by a fluorescence method as described by the assay manufacturer. The fluorescence of each sample was normalised to mg of tissue. Measurements were made at baseline, 30 and 45 mins of incubation, and the result is expressed as change from baseline.

### LPL promoter Methylation

100 ng of genomic DNA was bisulfite treated using the BisulFlash DNA modification kit (Epigentek, USA). Primers for bisulfite-converted DNA were designed with the online tool Methprimer (www.urogene.org//methprimer) covering 4 CpG sites in the LPL promoter region from bp −236 to −46 prior to the transcription start site of the LPL promoter. Primer sequences: left primer 5′-TGAGGGAGGATTGTAAGTGATAAATA, right primer 5′- CCCTATCTAAACACCAAACACAAAT. 20 ng bisulfite-treated DNA was PCR amplified with 1 cycle at 95 °C for 10 min, 40 cycles of 95 °C for 30 sec, 55 °C for 40 sec and 72 °C for 60 sec, followed by 1 cycle of 7 min at 72 °C. The PCR products were then supplied to a sequencing facility (AGRF, St Lucia QLD Australia) for capillary sequencing. Sequencing results were analysed with the online software tool for bisulfite-treated DNA BiQ (**biq**-analyzer.bioinf.mpi-inf.mpg.de/) giving the proportion of methylated and unmethylated residues at each CpG site.

### Statistical analysis

Experiments were performed in duplicate. Data are presented as mean +/− SEM unless stated otherwise. Differences between groups were examined with two tailed Mann–Whitney U tests (Prism version 5.03 software (GraphPad, La Jolla, CA)). Significance was set at < 0.05. Correlation analysis was performed with Spearman’s rho testing. Sensitivity analyses were performed excluding the data from the women with SGA infants and no difference was seen from the presented results.

## Results

### Study participants

Placentae from 16 women with preeclampsia (PE) and 20 normotensive (control) women were collected. This study includes women with late onset rather than early onset (pre 32 weeks gestation) PE. The women were matched for maternal BMI and gestational age of delivery. Maternal and pregnancy characteristics are shown in Table 2. There were 4 infants (25 %) born to women with PE who were small for gestational age infants (SGA). The mean gestational age of delivery did not differ between groups. The earliest delivery in women with PE was at 35.1 weeks and in the control group was 36.6 weeks.

**Table 2:** Clinical characteristics

	Control	PE	P
n	20	16	
Maternal age (years)	32.6 (1.0)	31.0 (1.6)	0.40
Maternal BMI in early pregnancy (mean(SD))	26.9 (1.5)	27.6 (1.6)	0.90
Caucasian ethnicity n (%)	19 (95)	16 (100)	1.0
Gestational age of delivery (weeks)	38.7 (0.2)	38.2 (0.4)	0.26
Birth weight (g)	3442 (86.84)	3056 (142.5)	0.06
Birth weight centile^a^	55.3 (7.2)	38.55 (7.60)	0.15
SGA n(%) ^b^	0	4 (25)	0.03
Infant sex (F/M)	9/11	8/9	1.0

^a^Adjusted birthweight centile [33] ^b^adjusted birth centile <10^th^

### ATGL

**Fig. 1:**
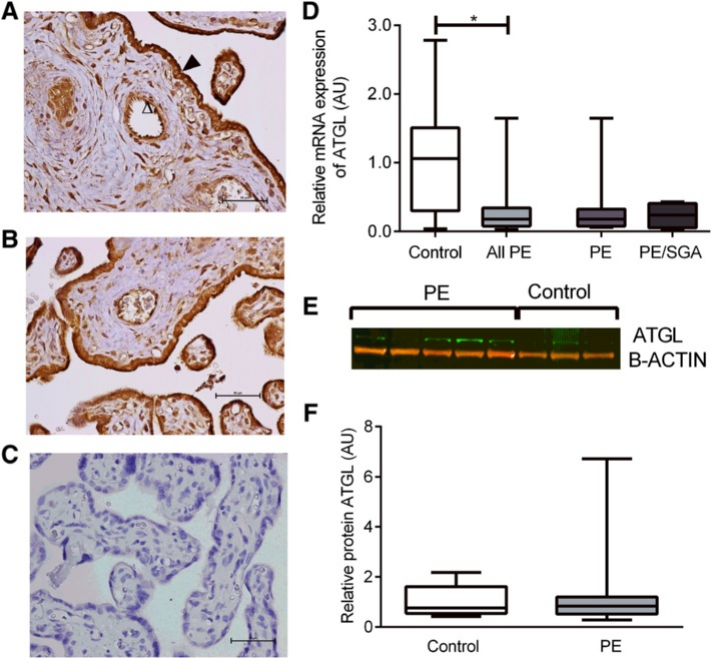
ATGL. **a** Immunohistochemisty for the detection of ATGL in control placenta, open arrow indicates endothelial cell staining, closed arrow indicates trophoblast staining. **b** immunohistochemistry in PE placenta, bar indicating 50 um. **c** negative control. **d** ATGL mRNA expression of control, all PE, PE alone and PE/SGA cases. **e** Representative Western blot result. **f** Relative protein expression of ATGL in control and PE placentae, boxes represent median with interquartile range, whiskers indicating 2.5–97.5 %, *, P < 0.05

ATGL was localized to syncytiotrophoblasts, endothelial cells and stromal cells including Hofbauer cells and decidual cells (Fig. 1a, b). The relative expression of ATGL mRNA was reduced in placenta from women with PE (PE median 0.15 AU (IQR 0.08–0.32) vs control (1.08 AU (0.30–1.57), P = 0.0003) (Fig. 1c). As the PE group included 4 women with SGA infants, we have also shown the PE alone and SGA alone results for mRNA expression in Fig. 1c. Given the smaller numbers, we did not perform statistical analyses on these different groups. There was no clear difference in the protein expression between placentae from control women or those with PE for ATGL (PE median 0.84 AU (IQR 0.52–1.20) vs control (0.77 AU (0.55–1.61), P = 0.98) (Fig. 1d, e).

### HSL

**Fig. 2:**
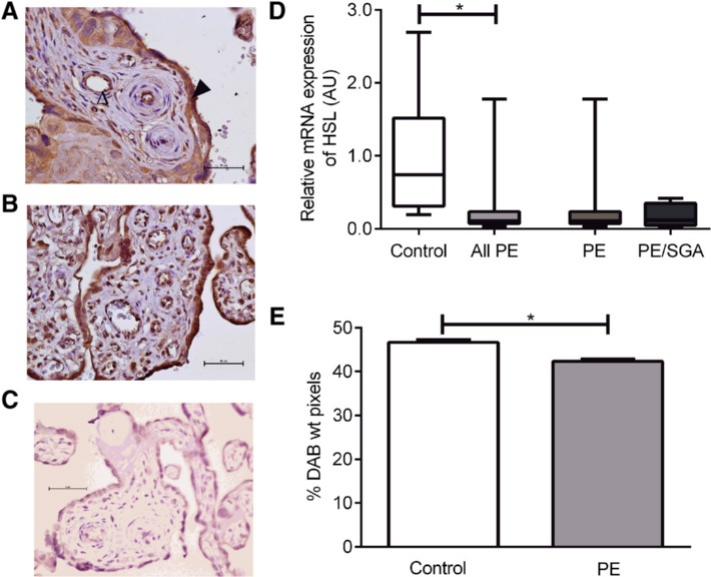
HSL. **a** Immunohistochemisty for the detection of HSL in control placenta, open arrow indicates endothelial cell staining, closed arrow indicates trophoblast staining. **b** immunohistochemistry in PE placenta. **c** negative control. **d** mRNA expression, showing control compared with all PE cases with the result of statistical analysis, as well as the result for PE alone or PE/SGA cases. **e** Relative protein expression as measured by semi-quantitative immunohistochemistry. *, P < 0.05

HSL was localized to syncytiotrophoblasts and endothelial cells but also to stromal cells including Hofbauer cells and decidual cells (Fig. 2a, b). The relative expression of HSL mRNA was reduced in placenta from women with PE (PE median 0.11 AU (IQR 0.06–0.15) vs control (0.83 AU (0.30–1.66), P < 0.0001) (Fig. 2c). HSL protein expression was assessed by quantitative immunohistochemistry. There was a reduction in HSL protein expression in placentae from women with PE compared to control (PE median 42.0 %DAB staining (IQR 36.5–48.7), Control 45.0 %DAB staining (39.7–53.6), P < 0.0001) (Fig. 2d).

### LPL and EL

**Fig. 3:**
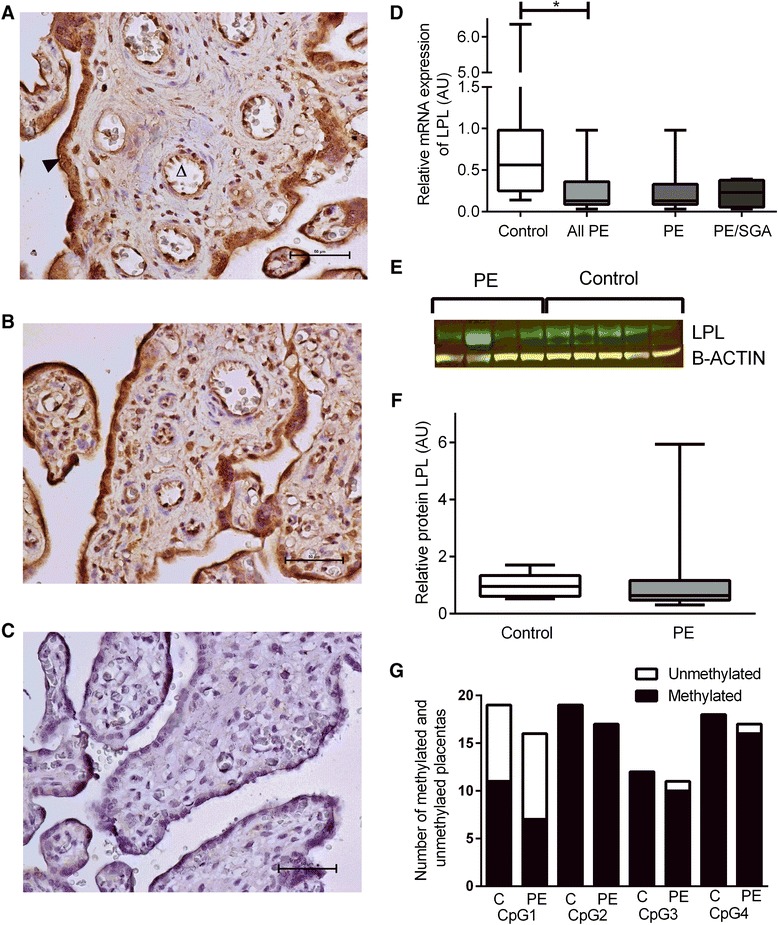
LPL. **a** Immunohistochemisty for the detection of LPL in control placenta, open arrow indicates endothelial cell staining, closed arrow indicates trophoblast staining. **b** immunohistochemistry in PE placenta. **c** negative control. **d** mRNA expression, showing control compared with all PE cases with the result of statistical analysis, as well as the result for PE alone or PE/SGA cases. **e** Representative Western blot result. **f** Relative protein expression, boxes represent median with interquartile range, whiskers indicating 2.5–97.5 %, *, P < 0.05. **g** LPL methylation analysis

**Fig. 4:**
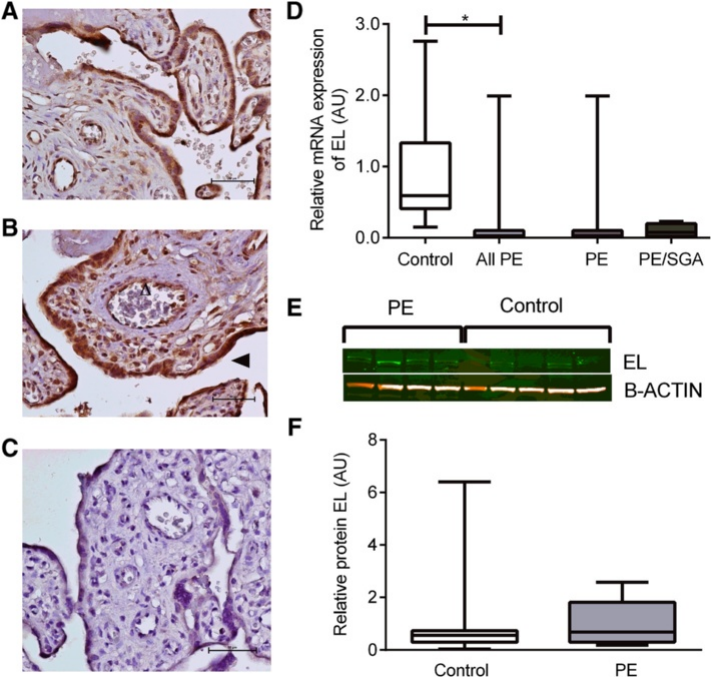
EL. **a** Immunohistochemisty for the detection of EL in control placenta, **b** immunohistochemistry in PE placenta, open arrow indicates endothelial cell staining, closed arrow indicates trophoblast staining. . **c** negative control. **d** mRNA expression, showing control compared with all PE cases with the result of statistical analysis, as well as the result for PE alone or PE/SGA cases. **e** Representative Western blot result, boxes represent median with interquartile range, whiskers indicating 2.5–97.5 %, *, P < 0.05. **f** Relative protein expression

The extracellular lipases LPL (Fig. 3a, b) and EL (Fig. 4a, b) were localized to syncytiotrophoblasts, endothelial cells and also to stromal cells including Hofbauer cells and decidual cells. The relative expression of LPL and EL mRNA were reduced in the placenta from women with late onset PE ((LPL: PE median 0.13 AU (IQR 0.08–0.33) vs control (0.57 AU (0.26–0.98), P < 0.0001) and EL: PE median 0.03 AU (IQR 0.01–0.09) vs control (0.68 AU (0.41–1.67), P < 0.0001)). There was no clear difference in the protein expression between placentae from control women or those with PE for LPL (PE median 0.63 AU (IQR 0.47–1.16) vs control (0.95 AU (0.61–1.33), P = 0.19) (Fig. 3e, f) or EL (PE median 0.68 AU (IQR 0.31–1.81) vs control (0.57 AU (0.30–0.74), P = 0.48) (Fig. 4d, e). DNA methylation of 4 CpG sites in the LPL promoter was investigated by bisulfite sequencing. While the levels of methylation varied between the CpG sites, there was no difference in the proportion of methylated vs unmethylated CpG sites in the LPL promoter between PE and control placentae (Fig. 3g).

### Lipase activity

**Fig. 5:**
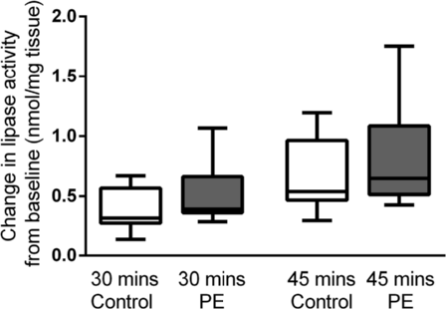
Lipase activity. Change in placental lipase activity taken at 30 and 45 mins, intissue from control pregnancies in white, and PE in grey. n = 8 control and n = 14 PE. Corrected to mg of tissue

Lipase activity was measured as fluorescence emission of hydrolyzed substrate in response to lipase enzyme activity and was tested in homogenized placental tissue samples. Lipase activity was measured at 30 and 45 minutes at room temperature (Fig. 5). There was no difference in placental lipase activity between PE and control pregnancies at 30 (PE median 0.39 (IQR 0.36–0.66) vs. control 0.32 (IQR 0.28–0.57), P = 0.12) or 45 minutes (PE median 0.65 (IQR 0.51–1.09) vs. control 0.53 (0.47–0.96), P = 0.35).

### Relationship with clinical factors

There was no relationship between the mRNA expression of any lipase and maternal early pregnancy BMI or infant birth weight for women with or without PE.

## Discussion

The current study, examining placentae from women with late onset PE and BMI matched women with uncomplicated pregnancies, has demonstrated reduced mRNA expression and reduced protein expression of HSL in placentae from women with PE. ATGL, LPL and EL showed reduced mRNA expression but unchanged protein expression. All lipases examined localized to the maternal and fetal sides of the placenta as well as the Hofbauer cells and decidual cells. There was no difference in localization between PE and control placentae for any of the four lipases described in this study, but we have demonstrated they are present in placental cells expected to be metabolically active.

The strengths of this study include a well characterized and matched cohort, however there are some limitations. For example, the HSL protein expression data in the current study needs to be assessed with some caution. We were unable to obtain western blot results for HSL, suggesting low levels of protein expression of HSL. Human HSL protein concentrations are high in adipocytes but much lower in other tissues such as skeletal muscle [29]. HSL mRNA is clearly present in placenta and HSL was localised with selective antibody by immunohistochemistry. Quantification of protein expression by image analysis is a validated technique, however, as with any analysis, it is possible that the reported reduction in HSL is a result of a type 1 error.

In late onset PE the fetus is usually well grown. The presence of a growth restricted fetus suggests that the PE is more severe or that another pathology underlies the growth restriction [30]. The inclusion of placentae from women with growth restricted infants in our study is therefore a potential confounder. However, we performed sensitivity analyses which did not change the direction or significance of any results indicating that the presence or absence of placentae related to growth restricted infants did not influence the results. Our samples were obtained from women with late onset PE only, which could have implications for applying our results to general PE. Early PE has been postulated to have quite different placental pathology and function to late PE [31, 30]. The findings of this study should therefore not be extrapolated to early PE, and the potential differences due to gestational age should be considered.

ATGL and HSL have not previously been examined in placentae from women with PE. We have recently shown that ATGL mRNA was increased and HSL mRNA decreased, with no difference in protein expression, in obese women with well controlled gestational diabetes mellitus compared to BMI matched controls [32]. ATGL and HSL are both intracellular lipases. They are involved in the mobilization of triacylglycerol from lipid droplets with ATGL mainly converting triacylglycerol to diacylglycerol and HSL converting diacylglycerol to monoacylglycerol. The decrease in ATGL and HSL mRNA and HSL protein seen here could result in reduced lipolysis of placental lipid droplets and hence reduced lipid transfer to the fetus.

In the current study, LPL mRNA expression was reduced in placentae from women with PE. However, there was no difference in protein expression or localisation. LPL mRNA expression has previously been found to be unchanged in PE [21] and unchanged [21] or increased in growth restricted infants [19, 20]. One reason for the disparity between our findings and the earlier studies with respect to growth restricted infants is the gestational age at delivery. In both the studies showing a reduced LPL mRNA expression in placentae from pregnancies with growth restricted infants, the placentae were from pregnancies delivered at a mean gestational age of 32 weeks at delivery, whereas delivery was at 39 weeks in the Laivouri study [21]. The mean gestational age at delivery of the placentae in our study is 38 weeks and the lack of change seen is consistent with the lack of change seen at the later gestation in the Laivouri study. The difference in gestational age rather than the condition itself could underlie the increase in LPL mRNA seen in the studies comparing 32 week placenta to term placenta.

EL mRNA expression was also reduced in placentae from women with PE, with no change in protein expression or localisation. EL expression has been previously found to be decreased in growth restricted infant associated placaentae compared with control [19]. It needs to be noted that this finding was in a cohort comparing term control and 32 week growth restricted infant related placentae, and that they reported an increase in placental EL expression from the first to third trimester. Once again, in our study, with careful matching of gestational age, EL protein expression was unchanged in PE.

The current study found no difference in overall lipase activity measured in placental biopsies. This is consistent with the results of a previous study in preterm and term pregnancies complicated by growth restricted infants that reported no change in overall placental triglyceride hydrolase activity but reduced LPL activity in isolated placental microvillous membrane [18]. In contrast, an older study showed greater LPL but lower intracellular lipase activity in placentae from PE and IUGR pregnancy [22]. The assay used in the current study is conducted at pH 7 and measures overall triglyceride hydrolase activity. The similar lipase activity levels we report are in keeping with the lack of change in protein levels for 3 of 4 lipases we examined, suggesting that in term PE, placental lipases are unaltered.

## Conclusion

The current study demonstrated a decrease in mRNA expression in all four lipases. A small decrease in HSL protein was seen, but no changes in protein expression for ATGL, LPL or EL were demonstrated. There was no difference in lipase activity in placentae from pregnancies complicated by late onset PE compared to control. This suggests that this aspect of placental lipid processing is not altered in late onset PE and does not underlie the differences seen in infant growth.
